# NDRG1 Expression Is an Independent Prognostic Factor in Inflammatory Breast Cancer

**DOI:** 10.3390/cancers12123711

**Published:** 2020-12-10

**Authors:** Emilly S. Villodre, Yun Gong, Xiaoding Hu, Lei Huo, Esther C. Yoon, Naoto T. Ueno, Wendy A. Woodward, Debu Tripathy, Juhee Song, Bisrat G. Debeb

**Affiliations:** 1 Department of Breast Medical Oncology, The University of Texas MD Anderson Cancer Center, Houston, TX 77030, USA; esschlee@mdanderson.org (E.S.V.); xhu7@mdanderson.org (X.H.); nueno@mdanderson.org (N.T.U.); dtripathy@mdanderson.org (D.T.); 2 Morgan Welch Inflammatory Breast Cancer Clinic and Research Program, The University of Texas MD Anderson Cancer Center, Houston, TX 77030, USA; yungong@mdanderson.org (Y.G.); leihuo@mdanderson.org (L.H.); wwoodward@mdanderson.org (W.A.W.); 3 Department of Pathology, The University of Texas MD Anderson Cancer Center, Houston, TX 77030, USA; ecyoon@mdanderson.org; 4 Department of Radiation Oncology, The University of Texas MD Anderson Cancer Center, Houston, TX 77030, USA; 5 Department of Biostatistics, The University of Texas MD Anderson Cancer Center, Houston, TX 77030, USA; jsong1@mdanderson.org

**Keywords:** NDRG1, N-myc downstream-regulated gene 1, IBC, inflammatory breast cancer, survival

## Abstract

**Simple Summary:**

Inflammatory breast cancer (IBC) is a rare and aggressive variant of breast cancer that is responsible for a significant number of breast cancer-related deaths. Herein, we describe how the expression of a specific protein named N-myc downstream-regulated gene 1 (NDRG1), commonly described as a gene that prevents the spread of cancer cells to distant organs, may have a paradoxical role in cancer progression in IBC. We found that the level of expression of NDRG1 in tumor tissues predicts the survival outcome of patients with IBC. We also observed that NDRG1, together with other important prognostic factors such as estrogen receptor status and stage, could be used to further analyze prognostic outcome or treatment response of patients.

**Abstract:**

NDRG1 is widely described as a metastasis suppressor in breast cancer. However, we found that NDRG1 is critical in promoting tumorigenesis and brain metastasis in mouse models of inflammatory breast cancer (IBC), a rare but highly aggressive form of breast cancer. We hypothesized that NDRG1 is a prognostic marker associated with poor outcome in patients with IBC. NDRG1 levels in tissue microarrays from 64 IBC patients were evaluated by immunohistochemical staining with NDRG1 (32 NDRG1-low (≤median), 32 NDRG1-high (>median)). Overall and disease-free survival (OS and DSS) were analyzed with Kaplan–Meier curves and log-rank test. Univariate analysis showed NDRG1 expression, tumor grade, disease stage, estrogen receptor (ER) status, and receipt of adjuvant radiation to be associated with OS and DSS. NDRG1-high patients had poorer 10-year OS and DSS than NDRG1-low patients (OS, 19% vs. 45%, *p* = 0.0278; DSS, 22% vs. 52%, *p* = 0.0139). On multivariable analysis, NDRG1 independently predicted OS (hazard ratio (HR) = 2.034, *p* = 0.0274) and DSS (HR = 2.287, *p* = 0.0174). NDRG1-high ER-negative tumors had worse outcomes OS, *p* = 0.0003; DSS, *p* = 0.0003; and NDRG1-high tumors that received adjuvant radiation treatment had poor outcomes (OS, *p* = 0.0088; DSS, *p* = 0.0093). NDRG1 was a significant independent prognostic factor for OS and DSS in IBC patients. Targeting NDRG1 may represent a novel strategy for improving clinical outcomes for patients with IBC.

## 1. Introduction

Inflammatory breast cancer (IBC) is one of the most aggressive forms of breast cancer. Although rare, accounting for only 1%–4% of newly diagnosed breast cancer cases, it is responsible for a disproportionately high 10% of breast cancer-related deaths in the United States [1,2]. IBC has a unique biology characterized by rapid proliferation and metastasis; indeed, almost all patients have lymph node involvement and more than 33% of patients with IBC present with distant metastasis at the time of diagnosis [3,4]. Even with multimodality treatment approaches that include systemic chemotherapy, surgery, and radiation therapy, the prognosis for patients with IBC is worse than for non-IBC patients (overall survival (OS) rates 40% versus 63% at 5 years) [5,6,7]. This may be due in part to 70% of IBC patients presenting with aggressive subtypes of HER2+ or triple-negative breast cancer (TNBC), compared with 40% of non-IBC tumors [8]. Efforts have been undertaken to identify molecular markers and therapeutic targets distinct to IBC and have identified important targets and pathways, including EGFR, E-cadherin, eIFG4I, RhoC, and TIG1/AXL [9,10,11,12,13]. However, no IBC-specific molecular signature or target has been identified thus far, and effective targeted therapies for this disease remain limited.

N-myc downstream-regulated gene 1 (NDRG1) is a stress response protein involved in hypoxia, cell growth, lipid metabolism, and resistance to chemotherapy [14,15,16,17,18,19]. NDRG1 is widely known as a metastasis suppressor in breast cancer, acting mainly by suppressing migration and invasion of breast cancer cells [20,21,22]. However, we and others have shown NDRG1 to be a tumor promoter in aggressive breast cancer [23,24,25]. Nagai and colleagues also showed that high expression of NDRG1 was associated with aggressive breast cancer behaviors, including the advanced stage at presentation and high-grade tumors and that NDRG1 was independently associated with poor survival outcome [26]. However, the expression of NDRG1 and its clinical importance in IBC remains unknown.

Herein, we examined the expression of NDRG1 by using immunohistochemical staining of a tissue microarray (TMA) composed of samples from IBC patients and evaluated the expression of NDRG1 and its correlation with survival outcomes. We also assessed the association between NDRG1 expression and outcome stratified by known prognostic factors. Our findings showed that high expression of NDRG1 in IBC tumors was an independent predictor of worse OS and disease-specific survival (DSS).

## 2. Results

**Figure 1:**
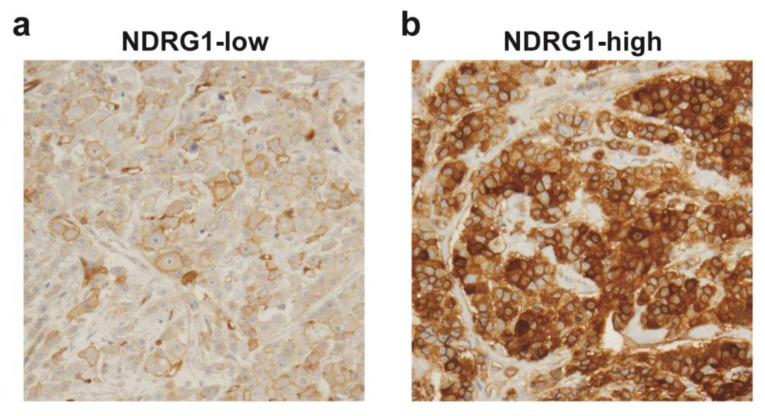
Immunohistochemical staining of N-myc downstream-regulated gene 1 (NDRG1) in inflammatory breast cancer (IBC) tumors. Representative images of NDRG1 immunostaining of (**a**) an NDRG1-low IBC tumor and (**b**) an NDRG1-high IBC tumor.

To determine whether NDRG1 protein expression is associated with outcome in IBC, immunohistochemical staining was performed on TMAs from 64 patients with primary IBC who were treated between 1991 and 2004 at The University of Texas MD Anderson Cancer Center. The tissues used to create the TMA were from refractory or residual IBC tumors after neoadjuvant systemic therapy. The average age of these patients was 50 years (range 23–75 years). Eighty-three percent of patients were stage III, 80% high grade, 62% were ER-negative tumors, and 67% of these patients received adjuvant radiation. The median follow-up time for the patients studied was 11.7 years, and the median OS time was 3.7 years. NDRG1 staining was predominantly cytoplasmic/membranous. Representative images of NDRG1-low and -high tumors are shown in Figure 1.

Table 1 summarizes patient characteristics based on NDRG1 expression status, NDRG1 expression was associated with negative HER2 status (*p* = 0.0077). Univariate analysis (Table 2) showed that NDRG1 expression (hazard ratio (HR) = 2.1, *p* = 0.0150), tumor grade (HR = 2.4, *p* = 0.0463), disease stage (HR = 4.6, *p* = 0.0011), ER status (HR = 0.4, *p* = 0.0098), and adjuvant radiation therapy (HR = 0.5, *p* = 0.0434) were associated with OS. The same variables were also associated with DSS (Table 2).

**Figure 2:**
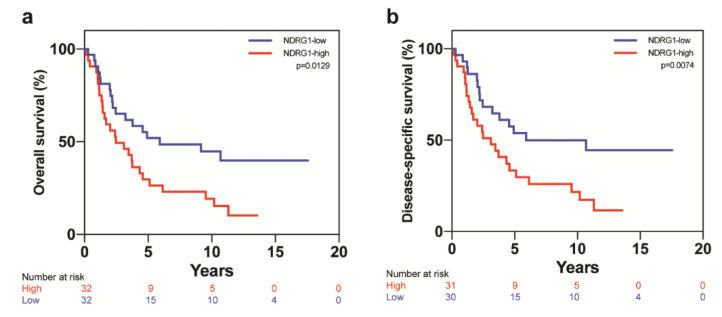
NDRG1 is a predictor of poor outcome in patients with IBC. Kaplan–Meier analysis showed that patients whose tumors had NDRG1-high expression had (**a**) worse overall survival and (**b**) worse disease-specific survival than did patients whose tumors had NDRG1-low expression.

The Kaplan–Meier method was used to evaluate the association of NDRG1 expression and survival over time. Patients with NDRG1-low tumors experienced better actuarial 10-year OS (*p* = 0.0129, Figure 2a) and DSS (*p* = 0.0074, Figure 2b). Patients with NDRG1-high tumors showed significantly lower 10-year OS and DSS rates than patients with NDRG1-low (OS, 19% vs. 45%, *p* = 0.0278; DSS, 22% vs. 52%, *p* = 0.0139). The median OS and DSS times were shorter for NDRG1-high patients (OS, 2.5 years; DSS, 3.1 years) than for NDRG1-low patients (OS, 5.9 years; DSS, 10.7 years). Multivariable model predictors of OS and DSS included NDRG1 expression, ER status, disease stage, and receipt of adjuvant radiation (Table 3). Tumor grade was identified as being associated with OS and DSS at the univariate level but not at the multivariable level. NDRG1-high expression was a strong independent predictor of OS (HR = 2.449, 95% confidence interval (CI) = 1.302–4.607, *p* = 0.0055) and DSS (HR = 2.727, 95% CI = 1.380–5.389, *p* = 0.0039).

**Figure 3:**
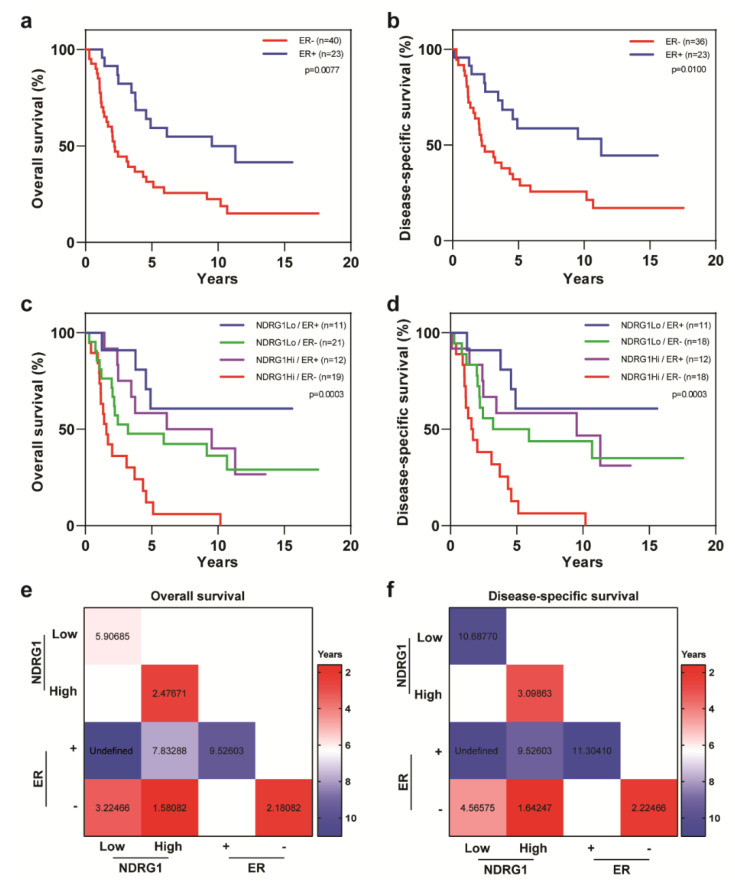
Overall survival and disease-specific survival in patients with IBC stratified by estrogen receptor (ER) status and NDRG1 expression. Patients with ER-negative tumors had (**a**) worse overall survival and (**b**) worse disease-specific survival versus patients with ER-positive tumors. (**c**,**d**) Stratification of patients by ER and NDRG1 expression status in terms of overall survival and disease-specific survival. Log-rank tests were used to obtain p values. (**e**,**f**) Median overall survival and disease-specific survival times, in years, for patients stratified by ER status and NDRG1 expression.

ER status was also an important prognostic factor for OS and DSS for patients with IBC; patients with ER-negative tumors had worse OS (*p* = 0.0077) and DSS (*p* = 0.01) relative to patients with ER-positive IBC tumors (Figure 3a,b). Multivariable analysis showed ER status to be an independent factor associated with OS (HR = 0.318, 95% CI = 0.158–0.641, *p* = 0.0014) and DSS (HR = 0.316, 95% CI = 0.151–0.665, *p* = 0.0024) (Table 3). Interestingly, NDRG1-high and ER-negative tumors were associated with the worst clinical outcomes for patients with IBC (OS, *p* = 0.0003; DSS, *p* = 0.0003; Figure 3c,d). Survival outcomes of ER-positive patients were not affected by NDRG1 expression (Figure 3c,d). Analysis of median OS and DSS times highlights the importance of stratifying patients for both variables: patients with ER-negative tumors had a median of 2.2 years for both OS and DSS, whereas those with ER-negative/NDRG1-high tumors had a median of 1.6 years, and ER-negative/NDRG1-low tumors had medians of 3.2 years OS and 4.6 years DSS (Figure 3e,f).

**Figure 4:**
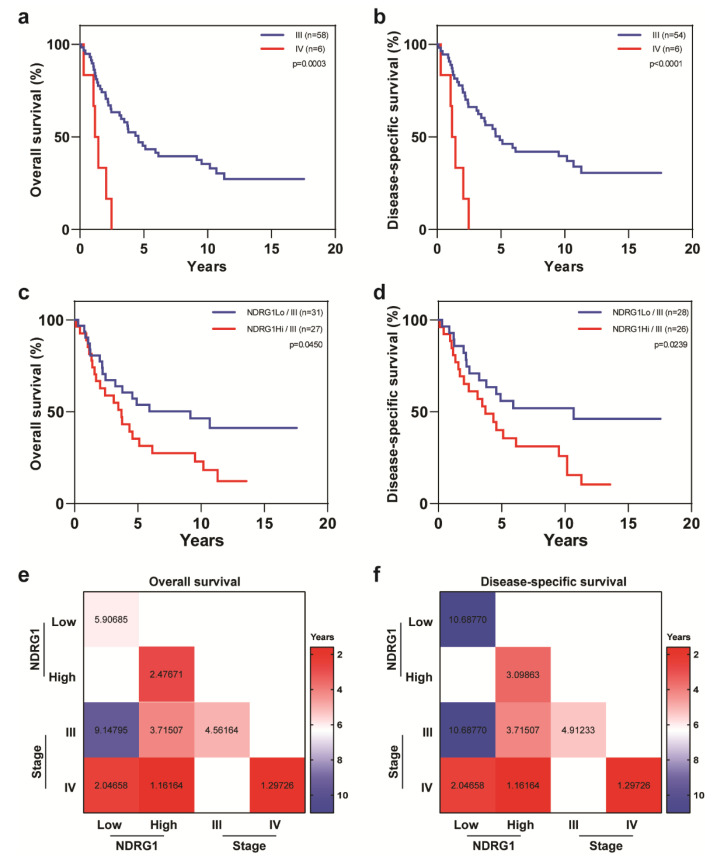
Overall survival and disease-specific survival in patients with IBC stratified by disease stage and NDRG1 expression. Patients with stage III IBC had better (**a**) overall survival and (**b**) disease-specific survival than did patients with stage IV IBC. (**c**,**d**) Patients with stage III IBC stratified by NDRG1 expression in terms of overall survival and disease-specific survival. Log-rank tests were used to obtain p values. (**e**,**f**) Median overall survival and disease-specific survival times, in years, for patients stratified by disease stage and NDRG1 expression.

Disease stage was another independent prognostic variable for OS (HR = 4.35, 95% CI = 1.685–11.229, *p* = 0.0024) and DSS (HR = 5.07, 95% CI = 1.908–13.473, *p* = 0.0024). Kaplan-Meyer analysis showed that patients with stage III IBC had better outcomes than did patients with stage IV tumors (OS, *p* = 0.0003; DSS, *p* < 0.0001) (Figure 4a,b). Further stratification of patients with stage III disease according to NDRG1 expression status showed a significant difference in outcomes, wherein patients with stage III NDRG1-high tumors had worse OS (*p* = 0.045) and DSS (*p* = 0.0239) than did patients with stage III NDRG1-low tumors (Figure 4c,d). We could not perform similar analyses of stage IV tumors owing to small patient numbers. Interestingly, the median OS times for patients with stage III tumors differed considerably by NDRG1 expression level, being 9.1 years in NDRG1-low tumors to 4.6 years for NDRG1-high stage III tumors (Figure 4e). Similarly, the median DSS times were 4.9 years for stage III NDRG1-low tumors and 10.7 years for stage III NDRG1-high tumors (Figure 4f).

**Figure 5:**
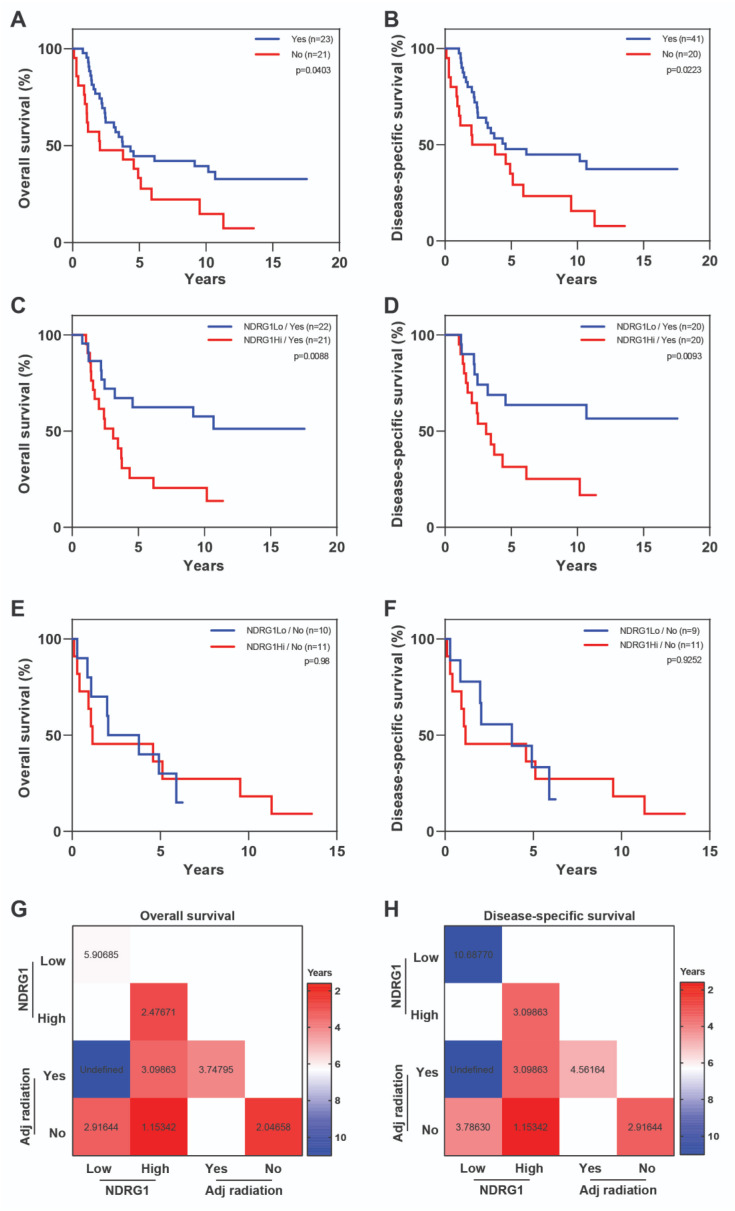
High NDRG1 expression correlated with worse outcomes among patients who received adjuvant radiation therapy. IBC patients who received adjuvant radiation had better (**A**) overall survival (**B**) and disease-specific survival than did patients who did not receive radiation. (**C**,**D**) Patients who received adjuvant radiation treatment stratified by NDRG1 expression in terms of overall survival and disease-specific survival. (**E**,**F**) Patients who did not receive adjuvant radiation stratified by NDRG1 expression in terms of overall survival and disease-specific survival. Log-rank tests were used to obtain p values. (**G**,**H**) Median overall survival and disease-specific survival times, in years, for patients stratified by NDRG1 expression and adjuvant radiation treatment status.

Receipt of adjuvant radiation was also an independent variable marginally related to DSS (HR = 0.575, 95% CI = 0.301–1.097, *p* = 0.0930) (Table 3). Patients who received adjuvant radiation had better survival outcomes than those who did not (OS, *p* = 0.0403; DSS, *p* = 0.0223) (Figure 5a,b). Among patients who received adjuvant radiation, those with NDRG1-high tumors showed poorer outcomes than those with NDRG1-low tumors (OS, *p* = 0.0088; DSS, *p* = 0.0128, Figure 5c,d). Among patients who did not receive adjuvant radiation therapy, NDRG1 expression did not correlate with survival outcomes (Figure 5e,f). The median survival times for all patients who received adjuvant radiation was 3.7 years for OS and 4.6 years for DSS. Stratification of radiation-treated patients by NDRG1 again showed distinct differences in survival time, with medians of 3.1 years for both OS and DSS for NDRG1-high tumors versus not achieved for NDRG1-low tumors (Figure 5g,h).

**Figure 6:**
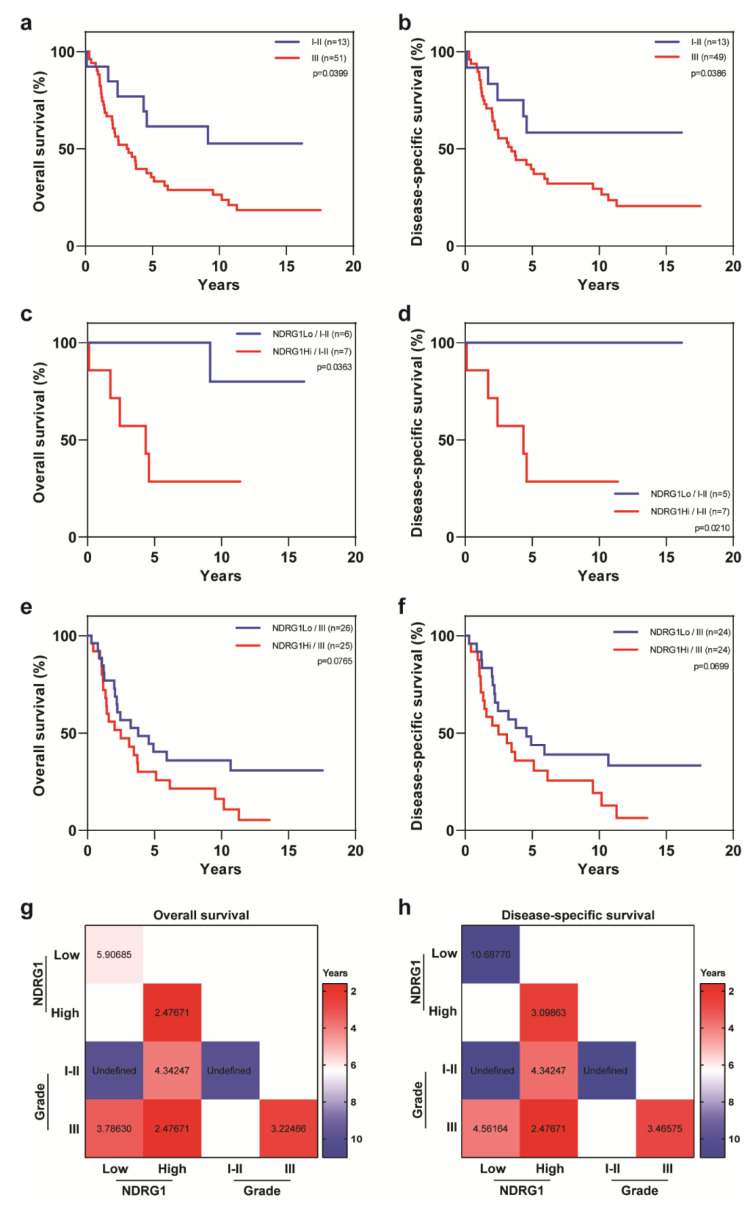
Overall survival and disease-specific survival in patients with IBC stratified by tumor grade and NDRG1 expression. Patients with IBC and low-grade cancer had better (**a**) overall survival and (**b**) disease-specific survival than did patients with grade III disease. (**c**,**d**) Patients with low-grade IBC stratified by NDRG1 expression in terms of overall survival and disease-specific survival. (**e**,**f**) Patients with high-grade IBC stratified by NDRG1 expression in terms of overall survival and disease-specific survival. Log-rank tests were used to obtain *p* values. (**g**,**h**) Median overall survival and disease-specific survival times, in years, for patients stratified by NDRG1 expression and tumor grade.

As expected, patients with lower tumor grades (I-II) had better outcomes than those with high-grade tumors (OS, *p* = 0.0399; DSS, *p* = 0.0386; Figure 6a,b). Despite the small number of low-grade tumors, we observed a significant difference in OS (*p* = 0.0363) and DSS (*p* = 0.0210) after stratifying for NDRG1-high versus NDRG1-low expression; patients with low-grade tumors and NDRG1-low expression had better outcomes than patients with NDRG1-high expression (Figure 6c,d). Outcomes may have been worse for patients with high-grade tumors and NDRG1-high expression relative to those with NDRG1-low expression, but those apparent differences were not statistically significant (OS, *p* = 0.0765; DSS, *p* = 0.0699) (Figure 6d,e). Low-grade, NDRG1-high tumors were associated with shorter survival, with median survival times of 4.3 years versus not achieved for low-grade, NDRG1-low tumors (Figure 6g,h).

## 3. Discussion

IBC remains a relatively poorly defined disease that lacks specific therapeutic targets and prognostic biomarkers; the molecular characterization of IBC could advance our understanding of its unique biology and provide opportunities that could be translated into novel therapeutic strategies to improve clinical outcomes. Herein, we report that NDRG1 protein expression was an independent predictor of poor survival outcomes for patients with IBC. In subset analyses, we report that NDRG1-high expression in patients with ER-negative, stage III tumors and patients who received adjuvant radiation had worse outcomes than did patients with NDRG1-low tumors. Our results suggest that IBC patients could be stratified not only by known prognostic markers but also by biological determinants such as NDRG1 expression status.

NDRG1 is a stress response gene that is highly activated and expressed in hypoxia and resistance to chemotherapy. Its function in breast cancer is widely described as a tumor and metastasis suppressor, acting mainly through inhibition of migration and invasion of cancer cells [20,21,22,27]. The induction of NDRG1 was shown in a mouse mammary tumor model to suppress metastasis by modulating WNT pathway signaling [21]. Chiang et al. also described how silencing NDRG1 expression in MCF-7 cells led to increased proliferation and invasiveness of those breast cancer cells [27]. Paradoxically, other studies showed that NDRG1 might function as an oncogene or a prognostic biomarker in aggressive forms of breast cancer [19,23,24,26]. Mao et al. found that NDRG1 could be used as a marker for invasive breast cancer, observing that NDRG1 expression was significantly higher in invasive breast cancer versus matched non-tumor tissues, and its levels were associated with progression from breast atypia to carcinoma. They also observed a correlation between advanced tumor stage and high NDRG1 expression [23]. Nagai et al. found an association between high NDRG1 expression and worse DSS and OS in a cohort of 600 patients: the 10 year OS rate was 35% for NDRG1-high versus 67% for NDRG1-low, and NDRG1 was an independent prognostic factor for both OS and DSS. Moreover, NDRG1 was expressed at higher levels in stage III and IV breast cancer and in grade 3 tumors [26]. More recently, a study by Sevinsky and colleagues observed similar results, wherein analysis of available data sets showed that patients with high expression of NDRG1 had worse recurrence- and metastasis-free survival. Moreover, they demonstrated that NDRG1 promotes breast cancer aggressiveness by altering lipid metabolism [19]. These observations are supported by our ongoing work showing that NDRG1 promotes tumorigenesis and brain metastasis in mouse models of aggressive breast cancer [25].

Expression of the ER is a well-known prognostic and predictive factor, and ER status is essential in the choice of treatment strategy. Patients with ER-positive breast cancer benefit from the use of hormonal therapy and have better OS than do patients with ER-negative disease, and this improvement is independent of disease stage and tumor grade [28,29,30]. Our results in the present study confirmed that ER status was, indeed, an independent factor related to both OS and DSS, with ER-negative IBC patients exhibiting worse clinical outcomes. ER-negative status is associated with aggressive growth and shorter survival. Interestingly, in our study, stratification of ER-negative patients by NDRG1 expression level showed significant differences in survival outcomes: ER-negative, NDRG1-high tumors were associated with worse outcomes than ER-negative, NDRG1-low tumors. However, no such difference was observed in ER-positive tumors stratified by NDRG1 expression. Our findings indicate that the clinical outcome of patients with ER-negative IBC can be stratified further based on NDRG1 expression status.

Adjuvant radiation therapy is an important part of breast cancer treatment and is known to improve breast cancer-specific survival and reduce tumor recurrence [31,32,33]. In the current study, we found that receipt of adjuvant radiation for IBC tumors marginally correlated with improved breast cancer-specific survival. We also showed that patients who received adjuvant radiation and had NDRG1 low-expressing tumors had better clinical outcomes than did those with NDRG1-high-expressing radiation-treated tumors. These hypothesis-generating findings suggest that the role of NDRG1 in local failure in breast cancer patients and radiation resistance warrants further investigation. Previous studies of rectal cancer cells have shown that NDRG1 is one of the top highly upregulated genes in response to ionizing radiation and that depleting it could be a promising strategy to sensitize cells to radiotherapy [34]. Many studies have been conducted to develop a “radiosensitivity signature” to stratify patients according to benefits from adjuvant radiation treatment [35,36,37]. However, no such molecular signature for radiation response has yet been identified.

This study has some limitations. First, the IBC TMA comprised tissues from refractory or residual tumors after neoadjuvant systemic therapy. Thus, the expression of NDRG1 described in this cohort may have been influenced by neoadjuvant chemotherapy. Further study that includes patients with pretreated IBC tumors is warranted to further refine these findings. The limited number of patient samples was another limitation of this study. More patient samples are needed to further validate our findings and conduct analyses of some important variables, such as loco-regional recurrence-free survival in patients who received radiation treatment stratified by NDRG1 expression status.

## 4. Materials and Methods

### 4.1. IBC Tumor Microarrays and Immunohistochemical Staining

This study was approved by the institutional review board of The University of Texas MD Anderson Cancer Center (LAB04–0821). Written informed consent was obtained from all patients prior to study enrollment. Details of disease diagnosis, preoperative and postoperative treatments, biomarker studies (including ER, PR, and HER2 status), and TMA construction with post-neoadjuvant residual tumors are reported elsewhere [38]. Briefly, patients received neoadjuvant chemotherapy, which was followed by mastectomy. Patients then received postmastectomy radiation to the chest wall and draining lymphatics (dose ranging from 60 to 71 Gy). For hormone receptor status (ER and PR), at least 10% of invasive cancer cells had to have nuclear staining to be considered positive, analyzed by immunohistochemical staining. HER2 was considered positive if at least 10% of invasive cancer cells had complete membranous staining in the IHC slide or had positive fluorescence in situ hybridization. For TMA construction, a manual tissue puncher was used to punch three cores of each formalin-fixed and paraffin-embedded post-neoadjuvant residual IBC tumors. Immunohistochemical staining of TMAs was done with a monoclonal antibody against NDRG1 (1:5000, #9485, Cell Signal) that was previously validated [39]. NDRG1 staining was evaluated by percentage (0%–100%) and intensity (weak, moderate and strong) of invasive tumor cells showing cytoplasmic and/or membranous staining. NDRG1 H-score was calculated by multiplying the percentage with intensity, and the NDRG1 H-score median (value of 120) was used as a cutoff, wherein 32 patients were grouped as NDRG1-low (≤median) and 32 as NDRG1-high (>median). Representative images of NDRG1-low and NDRG1-high tumors are shown in Figure 1. Supplementary file 1 contains the individual data for all variables analyzed and NDRG1 H-score values for each patient.

### 4.2. Statistical Analysis

Patient characteristics were summarized by NDRG1 value (low [≤median] vs. high [>median]) and compared between patients with NDRG1-low and patients with NDRG1-high tumors. Two-sample t-tests or Wilcoxon rank-sum tests were used for the comparison of continuous variables. Chi-squared tests or Fisher’s exact tests were used for the comparison of categorical variables. OS was defined as the interval from diagnosis to death, and DSS as the interval from diagnosis to death from breast cancer. Those patients without an event (death or breast cancer death) were censored at the last follow-up. Kaplan–Meier curves and log-rank tests were used to compare survival distributions. Univariate and multivariate Cox proportional hazards regression models were used to compare OS (and DSS) between NDRG1-low and -high groups, adjusting for other covariates. The proportional hazards assumption was checked by scaled Schoenfeld residual plots and correlation between the scaled Schoenfeld residuals and survival time. P values of < 0.05 indicated a statistically significant difference. SAS 9.4 (SAS Institute Inc, Cary, NC) was used for data analysis.

## 5. Conclusions

We are the first to show that NDRG1 expression was an independent prognostic factor for worse survival outcomes in patients with IBC, and together with other important prognostic factors, such as ER status and disease stage, can be used to further stratify prognostic outcome or treatment response in refractory tumors. Our findings suggest that targeting NDRG1 may provide a novel therapeutic strategy to improve outcomes for patients with IBC.

## Figures and Tables

**Table 1:** Clinical and pathologic characteristics of tumor samples from patients with IBC according to NDRG1 expression.

Covariate	Level	NDRG1-Low (*n* = 32)	NDRG1-High (*n* = 32)	*p*-Value
Age		51.5 ± 12.1	48.6 ± 12	0.3861
Race	Non-white	8 (25%)	5 (16%)	0.5356
	White	24 (75%)	27 (84%)	
Histologic type	Others	2 (6%)	4 (13%)	0.6719
	Ductal	30 (94%)	28 (87%)	
Grade	1–2	6 (19%)	7 (22%)	0.7560
	3	26 (81%)	25 (78%)	
Lymphovascular invasion	No	4 (14%)	3 (10%)	0.7065
	Yes	25 (86%)	27 (90%)	
Stage	III	31 (97%)	27 (84%)	0.1961
	IV	1 (3%)	5 (16%)	
Estrogen receptor	No	21 (66%)	19 (61%)	0.7209
	Yes	11 (34%)	12 (39%)	
Progesterone receptor	No	24 (75%)	19 (61%)	0.2425
	Yes	8 (25%)	12 (39%)	
HER2	No	12 (37%)	22 (71%)	0.0077
	Yes	20 (63%)	9 (29%)	
Triple-negative breast cancer	No	27 (84%)	20 (64%)	0.0879
	Yes	5 (16%)	11 (36%)	
Adjuvant radiation	No	10 (31%)	11 (34%)	0.7901
	Yes	22 (69%)	21 (66%)	

**Table 2:** Univariate Cox regression analysis on overall survival and disease-specific survival among patients with IBC.

		Overall Survival			Disease-Specific Survival		
Covariate	Level	HR	95% CI	*p*-Value	HR	95% CI	*p*-Value
Age	1 Unit Change	1.007	0.980–1.035	0.6080	1.000	0.971–1.029	0.9904
NDRG1	Low	1.000			1.000		
	High	2.107	1.155–3.842	0.0150	2.354	1.235–4.485	0.0092
Race	Non-white	1.000			1.000		
	White	1.823	0.769–4.321	0.1724	1.940	0.757–4.973	0.1676
Histologic type	Others	1.000			1.000		
	Ductal	1.027	0.368–2.870	0.9591	1.153	0.410–3.243	0.7874
Grade	1–2	1.000			1.000		
	3	2.404	1.014–5.698	0.0463	2.612	1.020–6.688	0.0453
Lymphovascular invasion	No	1.000			1.000		
	Yes	1.684	0.601–4.717	0.3216	1.493	0.529–4.213	0.4486
Stage	III	1.000			1.000		
	IV	4.638	1.847–11.647	0.0011	5.485	2.138–14.069	0.0004
Estrogen receptor	No	1.000			1.000		
	Yes	0.414	0.212–0.808	0.0098	0.426	0.211–0.860	0.0173
Progesterone receptor	No	1.000			1.000		
	Yes	0.699	0.359–1.361	0.2919	0.816	0.412–1.616	0.5598
HER2	No	1.000			1.000		
	Yes	0.748	0.409–1.366	0.3445	0.602	0.313–1.161	0.1300
Triple-negative breast cancer	No	1.000			1.000		
	Yes	1.557	0.810–2.992	0.1844	1.692	0.852–3.358	0.1328
Adjuvant radiation	No	1.000			1.000		
	Yes	0.538	0.295–0.982	0.0434	0.486	0.259–0.914	0.0252

**Table 3:** Multivariate Cox regression analysis on overall survival and disease-specific survival among patients with IBC.

		Overall Survival			Disease-Specific Survival		
Covariate	Level	HR	95% CI	*p*-Value	HR	95% CI	*p*-Value
NDRG1	Low	1.000			1.000		
	High	2.449	1.302–4.607	0.0274	2.727	1.380–5.389	0.0039
Estrogen receptor	No	1.000			1.000		
	Yes	0.318	0.158–0.641	0.0014	0.316	0.151–0.665	0.0024
Stage	III	1.000			1.000		
	IV	4.350	1.685–11.229	0.0024	5.070	1.908–13.473	0.0011
Adjuvant radiation	No	1.000			1.000		
	Yes	0.620	0.336–1.145	0.1269	0.575	0.301–1.097	0.0930

## Supplementary Materials

The following are available online at https://www.mdpi.com/2072-6694/12/12/3711/s1, Supplementary file 1: Individual data of the patients for all variables analyzed.
